# ‘The trial is owned by the team, not by an individual’: a qualitative study exploring the role of teamwork in recruitment to randomised controlled trials in surgical oncology

**DOI:** 10.1186/s13063-016-1341-1

**Published:** 2016-04-26

**Authors:** Sean Strong, Sangeetha Paramasivan, Nicola Mills, Caroline Wilson, Jenny L. Donovan, Jane M. Blazeby

**Affiliations:** Centre for Surgical Research, School of Social and Community Medicine, University of Bristol, Canynge Hall, 39 Whatley Road, BS8 2PS Bristol, UK; Division of Surgery, Head and Neck, University Hospitals Bristol NHS Foundation Trust, Bristol, UK

**Keywords:** Recruitment to randomised controlled trials, Qualitative research methods, Teamwork

## Abstract

**Background:**

Challenges exist in recruitment to trials involving interventions delivered by different clinical specialties. Collaboration is required between clinical specialty and research teams. The aim of this study was to explore how teamwork influences recruitment to a multicentre randomised controlled trial (RCT) involving interventions delivered by different clinical specialties.

**Methods:**

Semi-structured interviews were conducted in three centres with a purposeful sample of members of the surgical, oncology and research teams recruiting to a feasibility RCT comparing definitive chemoradiotherapy with chemoradiotherapy and surgery for oesophageal squamous cell carcinoma. Interviews explored factors known to influence healthcare team effectiveness and were audio-recorded and thematically analysed. Sampling, data collection and analysis were undertaken iteratively and concurrently.

**Results:**

Twenty-one interviews were conducted. Factors that influenced how team working impacted upon trial recruitment were centred on: (1) the multidisciplinary team (MDT) meeting, (2) leadership of the trial, and (3) the recruitment process. The weekly MDT meeting was reported as central to successful recruitment and formed the focus for creating a ‘study team’, bringing together clinical and research teams. Shared study leadership positively influenced healthcare professionals’ willingness to participate. Interviewees perceived their clinical colleagues to have strong treatment preferences which led to scepticism regarding whether the treatments were being described to patients in a balanced manner.

**Conclusions:**

This study has highlighted a number of aspects of team functioning that are important for recruitment to RCTs that span different clinical specialties. Understanding these issues will aid the production of guidance on team-relevant issues that should be considered in trial management and the development of interventions that will facilitate teamwork and improve recruitment to these challenging RCTs.

**Trial registration:**

International Standard Randomised Controlled Trial Number (ISRCTN): ISRCTN89052791.

## Background

Well-designed and well-conducted randomised controlled trials (RCTs) generate robust evidence to inform clinical and health-policy decision-making but many trials face problems with recruitment [1–4]. The recruitment process itself is often complex and involves several linked activities performed by clinical and research staff within and between different centres. Specific challenges in the recruitment process occur where the trial involves treatments routinely delivered by more than one clinical specialty (e.g. oncology treatment versus surgery) [5, 6].

There may be several reasons why trials of treatments delivered by more than one clinical specialty are difficult. The practicalities and co-ordination of balanced information provision for patients about both treatments can be challenging. Whilst clinicians may be comfortable explaining interventions they routinely deliver they may well be less confident conveying the effectiveness of treatments outside their specialist remit [7, 8]. The order in which eligible patients routinely consult with different clinical specialties within such trials may also have subtle influences on patient treatment preferences and, therefore, enrolment. In addition there is a need for these clinical teams to liaise closely with research professionals conducting the trial. The interdependency and complexity of components of the recruitment process means that teamwork within and between clinical and research teams is likely to be a factor in effective trial recruitment.

Multidisciplinary team (MDT) working within the delivery of cancer care is well-established and it is now mandatory in many countries for management decisions for all new patients with cancer to be made within MDTs [9–14]. MDTs bring together healthcare professionals from many different backgrounds, such as surgeons, oncologists and specialist nurses who, as well as agreeing to treatment strategies, share a responsibility for recruiting patients to trials. MDTs have been suggested as the ideal setting to help co-ordinate and maximise clinical trial recruitment but have to date not been evaluated with regard to this outcome [15, 16].

The importance and benefits of teamwork within and between clinical, multidisciplinary and research teams in recruitment to trials with interventions delivered by different clinical specialties is currently unknown. We undertook qualitative research to explore these issues within a multicentre feasibility trial in surgical oncology involving two diverse interventions delivered by oncologists or surgeons.

## Methods

### Study setting

This study was devised to explore the importance of teamwork in recruitment to a multicentre study in surgical oncology. The present study was nested within a feasibility RCT that aimed to establish whether a full trial comparing a surgical (oesophagostomy) with a non-surgical treatment (definitive chemoradiotherapy) for localised oesophageal squamous cell carcinoma (SCC) was viable [17]. This trial was chosen as the setting for the exploration of the role of teamwork in recruitment because of the diverse nature of the two interventions and because there were substantial variations in the number of patients successfully recruited between participating centres. Ethical approval was obtained for the present study and the feasibility study from the North Somerset and South Bristol Research and Ethics Committee (09/H0106/69).

### Description of the trial recruitment pathway and teams

Eligible patients were offered separate consultations with a member of the surgical team initially, and then with a member of the oncology team, to discuss the trial and treatment options. Centres recruited patients to the trial for different lengths of time between April 2010 and March 2013. During this time, 375 patients with oesophageal SCC were discussed in 331 MDT meetings, and 42 (11 %) patients were considered eligible. Of these, five patients were successfully randomised, all from a single centre (centre 3) (Table 1).

**Table 1:** Number of patients who were discussed, found to be eligible, and randomised at each centre

	Centre 1	Centre 2	Centre 3
Patients with oesophageal SCC discussed at MDT	162	50	163
Eligible for the pilot RCT	15	3	24
Randomised into pilot RCT	0	0	5

*MDT* multidisciplinary team, *RCT* randomised controlled trial, *SCC* squamous cell carcinoma

We aimed to interview healthcare professionals who participated in any aspect of recruitment to this study. This included members of the surgical team (consultant surgeons and specialist nurses), members of the oncology team (consultant oncologists) and members of the research team (research nurses, the chief investigator, the principle investigators and clinical trials co-ordinators).

### Data collection

Participants were purposively sampled to ensure that a range of different healthcare professionals across all of the study centres was included (Table 2). Interviewees in centre 3 were known to SS but not to CW. Interviewees in centres 1 and 2 were not known to either SS or CW. Interviews in centre 3 were conducted by CW and in centres 1 and 2 by SS. Interviews were conducted either face-to-face or over the telephone between April 2012 and March 2013. All participants consented to being interviewed. A semi-structured topic guide was developed from the literature on trial recruitment and healthcare team effectiveness to explore perceptions of teamwork and its role in recruitment to trials. The topic guide was modified iteratively throughout the study to reflect emergent findings (Appendix 1).

**Table 2:** Participant profile

Study ID	Centre	Job title
P1	1	Consultant oncologist
P2	1	Consultant oncologist
P3	1	Research nurse
P4	1	Clinical trials co-ordinator
P5	1	Consultant surgeon, Principle investigator
P6	2	Consultant oncologist, Principle investigator
P7	2	Consultant oncologist
P8	2	Consultant surgeon
P9	2	Consultant surgeon
P10	2	Research nurse
P11	2	Research nurse
P12	3	Consultant surgeon
P13	3	Consultant surgeon
P14	3	Consultant surgeon
P15	3	Consultant surgeon
P16	3	Consultant surgeon, Chief investigator
P17	3	Consultant oncologist
P18	3	Research nurse
P19	3	Research nurse
P20	3	Research fellow
P21	3	Specialist UGI nurse

*UGI* Upper gastro-intestinal

### Data analysis

Interviews were audio-recorded and transcribed verbatim, with identifying details anonymised. Thematic analysis was undertaken using the constant comparison technique of grounded theory [18, 19]. This aims to generate new hypotheses about phenomena that are derived or grounded in the data. Its central principle is of constant comparison, where new findings are systematically compared with existing data. In this way, similarities and differences can be identified and emerging theories refined through the ongoing assimilation of data. The computer software package NVivo 10 was used to support data storage and analysis. Transcripts were coded with key words or phrases and scrutinised for common themes. Identified themes were compiled into a coding frame, against which themes emerging from subsequent transcripts were compared. Coding was carried out by SS following initial double-coding with SP and inconsistencies were discussed and resolved with reference to the raw data. Emergent themes were further discussed and refined by consultation between SS, CW, SP, NM and JD. SS was relatively new to qualitative research but was well-supported by SP and NM, both of whom have a wealth of qualitative research experience. CW is an experienced qualitative researcher. JD, who has extensive qualitative research experience, oversaw the research. Sampling, data collection and analysis were undertaken iteratively and concurrently until data saturation, the point at which no new themes occurred, was considered to have been achieved.

## Results

### Participants

Twenty-four healthcare professionals were contacted via email and sent a study protocol explaining the study and asking them for an interview. Twenty-one responded, all of whom were interviewed. This included eight surgeons (including the chief investigator and a principle investigator), five oncologists (including a principle investigator), five research nurses, a specialist upper gastro-intestinal nurse, one research fellow, and the clinical trials co-ordinator (Table 1). The mean length of interview time was 30 min (range 10–53 min). Identified factors that influenced how team working impacted upon trial recruitment were centred on: the multidisciplinary team meeting, leadership of the trial, and the recruitment process. These themes are explained in the following sections with illustrative quotes.

### 1. The multidisciplinary team meeting

Surgeons, oncologists and specialists nurses across all centres described the weekly multidisciplinary team meeting as an important factor in recruitment. The MDT meeting was reported as an essential forum to discuss the study in general, such as ensuring eligibility criteria were clear and understood and also as an opportunity to ensure that all patients were screened for eligibility and not missed:

P14: I think it’s (the MDT meeting) a very good way of trying to get the information out that this trial is occurring and we will discuss every patient that comes through and it’s a very good way of flagging up actually they’re potentially suitable for recruitment to this trial. (Consultant surgeon**,** centre 3)

P2: I think (the MDT meeting) works well as a team, and it’s partly that there is no way that a patient could be missed so you can be absolutely sure that for this sort of study all the patients get considered and that’s the first step. (Consultant oncologist, centre 1)

#### Structure of the MDT meeting

The structure of the multidisciplinary team meeting differed between sites and this was described as having an important influence on teamwork and recruitment. In centres 1 and 3, all healthcare professionals involved in the study (surgeons, oncologists, specialist nurses and research nurses) attended one central MDT meeting. This appeared to reduce professional barriers as the study was perceived as part of the MDT team’s responsibility instead of the study team and clinical MDT team being viewed as two separate entities. Decisions regarding the eligibility of a patient were described as being made by the MDT and not by any one individual. This shared responsibility ensured ‘buy-in’ from all healthcare professionals involved:

P17: We have a large trial portfolio and we try to have ‘buy-in’ from the whole team to a clinical trial so the clinical trials that we do are agreed by the whole team then, not done in isolation. And the MDT decision will be to offer a clinical trial. That’s one of the reasons why we’ve been very successful in terms of actually putting people into trials because there is ‘buy-in’ from the whole team to do clinical research. So, I think it was an MDT decision that they were eligible for the study and we went from there**.** (Consultant oncologist, centre 3)

P14: No. I don’t see it as a surgical trial or an oncological trial. I see it as a team trial. I see it as an MDT trial. (Consultant surgeon, centre 3)

By contrast, in centre 2, rather than one central MDT meeting, several smaller hospitals had their own MDT meetings where patients with cancer were initially discussed. Any patients that were appropriate for potentially curative treatment (and, therefore, eligible for the study) were then also discussed at a central MDT meeting attended by clinicians from these peripheral hospitals. This occurred at another hospital location. The peripheral MDT structure was described by all interviewees in this centre as problematic. Each of the peripheral sites acted as an individual team meaning that there was not the same MDT *‘*buy-in’ as was described in centres 1 and 3, with communication limited by institutional and specialty boundaries. Lack of integration of the research nurses into the multidisciplinary team also appeared to be affected by this structure, with interviewees suggesting that integration would have been desirable and that the lack of it may have been a factor affecting recruitment:

P6: We have evolved, um, into a team which has maintained local routes, so we still have a system where we have three local MDTs where we do the diagnostics and the palliative treatments and then they come together, the only time we all come together is for potentially curative patients. By and large patients are managed initially by local teams and, therefore, for this study the big challenge was when the patients are first diagnosed then seen by a surgeon, the conversation evolves about what the treatment options are. So, by the time we were having the main decision around treatment, patients had either missed consultations in terms of sort of getting them recorded or they’d already evolved into whether they were keen on surgery or not keen on surgery… I’d have sort of six conversations with different teams and you know making sure everybody was on board, it was hard. (Consultant oncologist, centre 2)

P10: It would have helped to have surgical leadership at the peripheral sites, we were separate, we needed someone to lead the surgeons. If we had a research nurse based in the peripheral hospital it would have brought the two groups together. (Research nurse, centre 2)

P10: We are an independent oncology site – we are not attached to the peripheral hospitals – so it was very difficult to have any research nurses input to support the surgeons. We were relying on the nurse specialists, who were a little bit protective and didn’t understand and had some fear about the trial. Not having a research nurse based at the peripheral sites was very difficult for them and the surgeons needed the support. Research is alien to the specialist nurses, they don’t understand about research, obviously it’s a totally different role… (They) were protective of their patients and fearful of the study. (Research nurse, centre 2)

#### Length of time as a multidisciplinary team and involvement with a RCT

The duration that clinical teams had worked together as a multidisciplinary team within their centre was felt to be a significant contributor to overall team functioning. Working with the same team members for a number of years was reported as increasing the efficiency of the team. The timing of involvement in this RCT also emerged as an important influence on teamwork, with individuals who felt involved with the study from the inception, and who perceived that they had participated in influencing the design, describing more engagement in the study (centre 1 and 3).

P17: So, I’ve been here for quite a few years now and we’ve worked as a team for many years and that’s how we’ve always worked and I think the team works quite successfully and has functioned quite well over many years. (Consultant oncologist, centre 3)

P5: As a multidisciplinary team we had discussed (the trial) for 14 months – a long time. All the team knew that it was something we wanted to do. (Consultant surgeon, centre 1)

P9: I think probably raising the awareness and bringing the study to the forefront of people’s minds more often was needed. Certainly I wasn’t really fully aware of what the trial involved and what its aims and outcomes were until I came to that meeting in (city)… after the trial had started**.** (Consultant surgeon, centre 2)

### 2. Trial leadership

All informants initially described a single named leader within their centre as having overall responsibility for all aspects of the trial including ensuring that eligible patients were appropriately screened at the MDT meeting and maintaining the profile of the study. In the only centre to randomise patients (centre 3), views regarding leadership appeared to change over the course of the study, with the study becoming viewed less as being led by an individual and more as a ‘team trial’, with all members of the MDT having responsibility for delivery:

P12: (The trial) has raised the awareness within our own MDT about the different options. It has stopped being (name of individual’s) trial, which is what it was called. There were the comments about (name of individual’s) project or, you know (name of individual’s) trial rather than the trial that we were looking at. I don’t know whether (name of individual’s) trial equalled pet project. That’s an interpretation I took when certain individuals would say it. I don’t know whether that was the case or not because I’ve never challenged anybody on that but that was an impression I got to begin with, um, and I think it changed with time. (Consultant surgeon, centre 3)

P17: No, I think – I hope the trial is owned by the team. I mean (name of individual) is the chief investigator, absolutely fine, but the concept of the trial is owned by the team – doing the trial is owned by the team not by an individual. The aim of clinical research is to improve patient care in its generality and, therefore, the whole team should have an interest in it. Teams that are proactive in research tend to have better quality outcomes as well. Fewer complaints and better outcomes, so there are good reasons for teams to be involved in research. If individuals were involved in research that’s absolutely fine, but it’s much more effective with ‘buy-in’ from the whole team. (Consultant oncologist, centre 3)

In the other centres in which a single named person continued to be viewed as the lead, the specialty of the leader was reported as having an influence on team engagement with the trial, with engagement being less problematic if the leader and team members were from the same speciality:

P1: I think we have very much a collaborative approach and (name of consultant) leadership style is, is very good, so he’s up there in the bow but the rest of us are rowing quite happily behind. (Consultant oncologist, centre 1)

P6: I guess then its about enthusiasm of the surgeons to really take it on. It wasn’t their trial, you know they weren’t against it but they probably weren’t as proactive as I was to make it work.

### 3. The recruitment process

#### Treatment preferences

All interviewees believed that oncologists and surgeons, who were responsible for describing the study interventions, favoured a particular treatment. This resulted in scepticism about whether the study treatments were being described to patients with equal enthusiasm. Preferences were described as subtle and not openly expressed. Surgeons tended to believe that other surgeons favoured surgery and oncologists favoured chemoradiotherapy. Oncologists also believed surgeons favoured surgery:

P16: I don’t know whether fundamentally (name of oncologist) thinks chemoradiotherapy’s a better treatment. I have suspicions he does, but I’ve no evidence for it. (Consultant surgeon, centre 3)

P7: I thought potentially, you know not all of them, but some of the surgeons may have felt that occasionally it was operable and that was preferable treatment. (Consultant oncologist, centre 2)

P14: Some of my colleagues in the unit would want to operate on absolutely everything they had the opportunity to operate on, and so might not emphasise the chemoradiotherapy bit quite as strongly. (Consultant surgeon, centre 3)

P3: But of course the surgeon is obviously more biased to surgery and the oncologist is biased to that treatment. I think subconsciously the way they talk to them… It may be something even more subtle than in words that they are saying. (Research nurse, centre 1)

In view of these biases one interviewee suggested that a member of the research team, as opposed to a surgeon or oncologist, would be better placed to provide a balanced description of the study and the involved treatment arms:

P13: You almost want the patients informed and consented and entered into the trial by a non-oncologist and non-surgeons. You want somebody who knows about both treatments, very well and in detail, knows about potential complications but also is removed from the frontline, so that they can impart as much information as possible in an unbiased way. (Consultant surgeon, centre 3)

The order in which patients had their treatment consultations (either with a surgeon first and then an oncologist, or vice versa) was believed to also influence treatment choices. Interviewees believed that patients were developing strong treatment preferences in line with the speciality of the clinician that they saw first and, therefore, declining randomisation. Possible solutions suggested to combat this bias included either changing the order in which they saw the specialists or to have a single joint consultation in which a surgeon and an oncologist both sit in the same consultation and explain the treatments to the patient:

P2: Well, certainly that’s our experience here, particularly with the way that we did the consultations is that they saw surgeons first and then oncologists. They would often come to me as the oncologist and say – you know I’ve already made my mind up – and they usually wanted surgery but it might be that they wanted radiotherapy. By the time they see the second clinician they already have quite firm views about which treatment they want. The other suggestion was that maybe we could be seeing the patients in a different order altogether or in a joint clinic. I think that would be interesting as then for everything the surgeon says the oncologist can give their opinion. You could sort of act as a double act to give the arguments for and against both mortalities of treatment. (Consultant oncologist, centre 1)

P13: I think the main problem of the trial was who saw them first. If the oncologist saw them first then they would be able to lay out their stall first – with the pluses and the minuses and then if they were seen again – in a few days’ time by the surgeons then foremost in their mind would be what the oncologist had to offer and we (as surgeons) would then have to, in inverted commas, compete with that. Whereas, if it happened the other way round we would then at least get our information across on a blank canvas. I think that’s the difficult thing with two very different treatments with two very different side-effect and complication profiles. (Consultant surgeon, centre 3)

## Discussion

This study has explored the importance of teamwork in recruitment to a multicentre study in upper gastro-intestinal cancer involving treatments delivered by different clinical specialties. The importance of the weekly MDT meeting to establish patient eligibility and for building a sense of a ‘team’ amongst the healthcare professionals was emphasised by team members. Shared leadership models across specialty and professional boundaries appeared to motivate enthusiasm for the study. Clinician bias towards the treatment routinely offered by their specialty was perceived by all interviewees. This led to scepticism about balanced information provision to patients which was believed to have a detrimental effect on recruitment rates. In light of these findings, it is recommended that trials consider the teams participating in recruitment and that future work develops interventions to facilitate ‘trial teams’ that span different clinical specialties.

A conceptual model known as ITEM (the Integrated Team Effectiveness Model) has been developed by Lemieux-Charles to aid the understanding of the effectiveness of teams in healthcare [20]. This model depicts the interactions between the task design, team process, and team effectiveness. Whilst useful to begin building theories, the authors acknowledge that more detailed and specific models are in need of development for different types of healthcare teams, such as those responsible for trial recruitment [20]. The value of the MDT meeting to trial recruitment has previously been described although these studies did not focus on challenging trials of diverse interventions [15, 16, 21]. A study examining MDT treatment recommendations at a single specialist upper gastro-intestinal MDT meeting showed that patients who were flagged up at the MDT meeting as potentially eligible for a trial were significantly more likely than those who were not to be screened for trial entry by the clinical trials nurses and consequently enrolled [16]. Shared decisions made in the context of the MDT meeting attended by all members in the team responsible for recruitment allows provision of a similar clinical message from all involved in the patients’ cancer journey. This is likely to enhance patients’ final understanding of clinical equipoise and, therefore, likelihood of randomisation. Shared decisions made by the MDT team regarding trial eligibility may also reduce the anxiety encountered by some healthcare professionals who feel a conflict between their role as advocate for the patient and recruiter [22]. This is particularly important in trials involving interventions delivered by more than one specialty as detailed knowledge of the interventions is required in order to decide that a patient would be suitable to undergo each of the treatments.

Interviewees in our study who had worked together for longer periods of time (centres 1 and 3) were more positive regarding the team’s perceived ability to recruit into the study. This finding is in keeping with a survey of 72 UK breast cancer teams which showed that the length of time a team had worked together had a significant influence on the ability to effectively co-ordinate the service [23]. A novel RCT investigated the influence of an educational intervention involving 22 cancer MDTs in the UK aimed at improving the awareness, involvement, communication, and recruitment rates to clinical trials [24]. Attendance by teams at the focused workshops improved several aspects of team functioning, such as team members’ awareness of their colleagues’ roles in regard to trial recruitment and time to develop specific recruitment strategies that work for the entire team as opposed to individual groups. However, this intervention failed to show a significant impact on the total number of patients approached regarding trial entry.

Whilst other studies have examined individual clinicians’ reasons for choosing not to enter potentially eligible patients into a trial or the willingness to counter expressed patient preferences, we are unable to find other published literature that has explored perceptions regarding a colleague’s treatment preferences [25, 26]. The majority of interviewees in our study believed that surgeons and oncologists had treatment preferences for the treatment that they were responsible for delivering (surgery or chemoradiotherapy respectively). This led to suspicions regarding whether the two treatments were being described to patients with equal enthusiasm. Perceived treatment preferences were not discussed between recruiters and appeared to be considered inevitable. It is possible that holding joint consultations in which the surgeon and oncologist sit together and explain the treatments and the study to the patient may reduce this problem or an independent research nurse could be trained to explain both treatments to patients to reduce these problems and improve recruitment. A nested study within the ProtecT trial (an RCT of treatments for localised prostate cancer) randomised patients to either a recruitment consultation with a consultant urologist or a research nurse, finding no significant difference in recruitment rates but a potential economic saving associated with research nurses compared to consultant surgeons undertaking the recruitment consultation [27].

Although this research is exploratory and novel, it is potentially limited because only one randomised trial has been studied and whether findings may be generalised to other settings requires further research. We did, however, purposively select participants to interview to achieve maximum variation within this study setting. Another possible limitation is that the majority of the interviews (centres 1 and 2) were conducted by a clinical academic with a background in surgery which potentially could have influenced the findings. To minimise this a topic guide was followed, interviews were double-coded and themes discussed between authors to ensure reliability of the analysis. Interviews at centre 3 were also conducted by a qualitative researcher who did not have a surgical background, with similar findings emerging. Triangulation of data would have also strengthened this research. This could have been achieved by either undertaking observations of the MDT meetings in order to directly observe different team dynamics or interviewing patients to explore the influences of teamwork on patients’ willingness to participate.

## Conclusions

This qualitative study has highlighted a number of aspects of team functioning that are important for recruitment to RCTs involving interventions delivered by different clinical specialties. Ongoing work will continue to examine the specific aspects of task design and team process that aid recruitment in other RCTs and develop interventions that will facilitate teamwork and improve future recruitment.

## Appendix 1

### Topic guide

**Oesophageal squamous cell cancer: induction chemotherapy and oesophagectomy versus induction chemotherapy and chemoradiotherapy – a feasibility study**

**Topic guide – Interviews exploring recruitment and teamwork**

### Introduction

1. Introduce yourself
2. Gain written consent (Consent Form 08)
3. Describe your title and role

### Background

1. Tell me about your involvement in the recruitment to trials, for example, the SCOPE trial. Probe for details.
2. Would you describe yourself as a scientist, clinician, doctor/nurse, trialist, researcher, or…? Or do you have more than one role? (If more than one role – are these roles complementary or are there conflicts?)
3. Have you received any training as a recruiter or for RCTs in general?
4. Tell me about your involvement in the recruitment to the SCC feasibility/chemoradiotherapy trial.
5. What is involved in the treatment arms of this trial?
6. When did recruitment start?
7. How well is recruitment going? Probe for details.
8. Do you think your colleagues (PIs and recruiters) are ‘in equipoise’? Probe for details.

### Recruitment pathway and teamwork

1. Are the eligibility criteria for the RCT clear? Do you think PIs agree with them (and comply with them)?
2. Are there reasons why recruitment is/would be difficult in this RCT, or have you had difficulties with recruitment?
3. Would you say that you are ‘in equipoise’? What does ‘equipoise’ mean to you?
4. How are patients recruited into this RCT, details form screening to randomisation and who is involved or mainly responsible for each step? Who is involved with recruitment?
5. Who undertakes the assessment of patient eligibility for this RCT? Are you involved in eligibility assessment?
6. Who has lead this project, views about leadership?
7. Relationships and communication with other members of multidisciplinary team, including the research nurses?
8. Do you ever have a feeling during an appointment that a patient should really have one treatment rather than another? Probe: Why? What do you do about that?

### Difficulties during recruitment

1. What would you say are the main difficulties that this trial faces with recruitment?
2. Is recruitment organised well? Do other people explain the RCT to patients?
3. Are there difficulties with any particular arm?
4. Do patients have strong preferences?
5. Do you think this RCT is the right thing for these patients?

### Personal views

1. Do you think this RCT will be successful? Probe all the reasons for answer.
2. Do you know (or have a hunch) about what the outcome of the RCT will be?
3. If you were a patient in this position today, would you agree to be recruited to the RCT and be randomised? Or would you choose a treatment? Which one?

### Finally – improving recruitment

1. What might, in your opinion, improve the recruitment appointments?

### Close the interview

1. Record baseline information on the interviewee (job, grade).
2. Thank the interviewee for participating in the interview.

## Abbreviations

MDT: multidisciplinary team
SCC: squamous cell carcinoma
UK: United Kingdom
